# Large-Scale Production of Lentiviral Vectors: Current Perspectives and Challenges

**DOI:** 10.3390/pharmaceutics12111051

**Published:** 2020-11-03

**Authors:** Eduardo Martínez-Molina, Carlos Chocarro-Wrona, Daniel Martínez-Moreno, Juan A. Marchal, Houria Boulaiz

**Affiliations:** 1 Biopathology and Medicine Regenerative Institute (IBIMER), University of Granada (D.M.), 18016 Granada, Spain; edumolinica@correo.ugr.es (E.M.-M.); cchocarro@ugr.es (C.C.-W.); daniel0mmd@ugr.es (D.M.-M.); jmarchal@ugr.es (J.A.M.); 2 Department of Human Anatomy and Embryology, University of Granada, 18016 Granada, Spain; 3 Excellence Research Unit “Modeling Nature” (MNat), University of Granada, 18016 Granada, Spain; 4 Biosanitary Institute of Granada (ibs.GRANADA), SAS-Universidad de Granada, 18016 Granada, Spain

**Keywords:** lentiviral vector, gene therapy, large-scale production, bioreactor

## Abstract

Lentiviral vectors (LVs) have gained value over recent years as gene carriers in gene therapy. These viral vectors are safer than what was previously being used for gene transfer and are capable of infecting both dividing and nondividing cells with a long-term expression. This characteristic makes LVs ideal for clinical research, as has been demonstrated with the approval of lentivirus-based gene therapies from the Food and Drug Administration and the European Agency for Medicine. A large number of functional lentiviral particles are required for clinical trials, and large-scale production has been challenging. Therefore, efforts are focused on solving the drawbacks associated with the production and purification of LVsunder current good manufacturing practice. In recent years, we have witnessed the development and optimization of new protocols, packaging cell lines, and culture devices that are very close to reaching the target production level. Here, we review the most recent, efficient, and promising methods for the clinical-scale production ofLVs.

## 1. Introduction

The main goal of gene therapy is to transfer a therapeutic gene into patients’ cells and achieve long-term expression to treat a disease. Since the first clinical trial in 1990, gene therapy has been used for many applications, mainly for treating monogenic diseases [1], as well as in cancer, as chimeric antigen receptor (CAR) T cell therapy does [2,3,4,5,6]. Since gene therapy began, more than 2500 clinical trials have been initiated, and different gene delivery vehicles or vectors have been employed for gene transfer [1,7]. Depending on the characteristics of the vectors, we can classify them into integrating and nonintegrating groups. Integrating vectors introduce foreign DNA into the cell genome, leading this DNA to replicate when the host cell divides. In nonintegrating vectors, foreign DNA remains in the nucleus in an episomal form [8].

The transduction process consists of delivering foreign genetic material into patients’ cells, and it can be carried out in vivo or ex vivo. Ex vivo gene therapy requires the extraction of patient cells; thus, subsequent vector transduction, which contains the gene of interest, occurs in the laboratory. After that, it is necessary to select and amplify the transduced cells to obtain the correct number to obtain a therapeutic outcome. Finally, the modified cells are reinfused into the patient. On the other hand, in vivo gene therapy consists of gene transfer directly into the patient in a particular region; thus, transduction occurs inside the patient.

Nowadays, viral vectors are widely used for gene transfer and each virus has unique features, which makes it suitable for specific treatment [9]. The U.S. Food and Drugs Administration (FDA) and the European Medicines Agency (EMA) have approved several gene therapies during recent years that use different viral vectors: the adeno-associated viral-vector-based Luxturna and Zolgensma (Novartis) have been approved for the in vivo treatment of inherited blindness and spinal muscular atrophy, respectively [10,11], and Glybera (UniQure) to treat lipoprotein lipase deficiency in vivo [12]; Imlygic (T-VEC), which is based on a herpex–simplex viral vector, has been employed to enhance the systemic antitumor immune response for treating melanoma [13]; the gammaretroviral-vector-based Strimvelis (GlaxoSmithKline) for treating adenosine-deaminase-deficient severe combined immune deficiency and Yescarta (Gilead) for treating large B-cell lymphoma [14,15,16]; and cytomegalovirus and vesicular stomatitis-virus-based vectors, among others, have been employed as vaccine carriers [17,18,19,20]. In this review, we focus on lentiviral vectors (LVs) whose use in clinical trials is increasing in recent years.

## 2. Lentiviral Vectors

Lentiviruses belong to a genus of the *Retroviridae* family. Lentiviruses are RNA-based and replicate thanks to a retrotranscription process, leading the genetic integration into a DNA host genome [21]. Nowadays, the most used LVs are based on human immunodeficiency virus (HIV). However, there are nonprimate lentiviruses, such as feline immunodeficiency virus (FIV) and equine infectious anemia virus (EIAV), with interesting features as well [22]. LVs are widely used for gene transfer in both basic and clinical research because of their many advantages: capability of infecting both dividing and nondividing cells, integration into the host genome with a long-term expression, and their ability to avoid integration near transcription start sites, unlike gammaretroviral vectors (RVs) [1,23]. However, LV integrates near the sites of active chromatin, this being in sites of active gene expression where they can disrupt genes. Disruption of tumor suppressor genes by LV integration could lead to cancer [24,25], but it bears a lower risk than RVs, and even lower if LV includes a synthetic chromatin insulator cassette [26,27].

**Figure 1:**
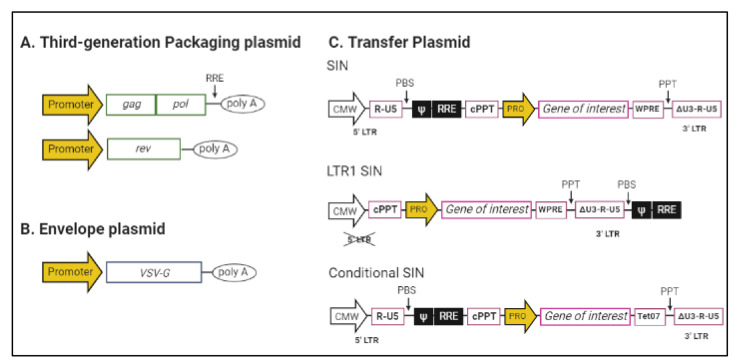
Plasmid designs for lentiviral vector (LV) production. (**A**) Third-generation LV has two separated packaging plasmids. (**B**) Vesicular stomatitis virus envelope glycoprotein (VSV-G) envelope protein is used for pseudotype LV to avoid cytotoxicity. (**C**) Transfer plasmids. Self-inactivating (SIN) plasmids to avoid long terminal repeat (LTR) promoter/enhancer activity. LTR1 reduces the total human immunodeficiency virus 1 (HIV-1) content. Conditional SIN: the plasmid expression is induced by the addition of doxycycline and cumate. Ψ: RNA packaging signal; ΔU3: SIN deletion in the U3 region of 3′ LTR; CMV: cytomegalovirus immediate-early promoter; cPPT: central purine tract; PBS: primer binding site; Poly A: poly (A) tail of multiple adenosine monophosphates; PPT: polypurine tract; Pro: internal promoter for transgene expression; RRE: rev response element; Tet07: tetracycline (Tet)-dependent regulatory system; U3-R-U5: U3, R and U5 regions from HIV-LTRs for efficient cleavage process; WPRE: woodchuck hepatitis virus post-transcriptional regulatory element.

The transformation of lentiviruses into viral vectors consists of separating the sequences needed for packaging and production from those encoding unnecessary viral proteins. First-generation HIV-based LVs separated the necessary elements of the virus into two plasmids. After several modifications, we are now at a mature and widely used third-generation of LVs (Figure 1), which are composed of four separate plasmids: two packaging plasmids *rev* and *gag-pol*, where (i) *rev* encodes protein expression for viral genome exportation and (ii) *gag-pol* express viral capsid structural proteins and the enzymes reverse transcriptase, integrase, and protease, respectively; (iii) an envelope plasmid encoding for glycoproteins, derived from the vesicular stomatitis virus envelope glycoprotein (VSV-G), which mediates the virus cell entry; and (iv) the transgene of clinical or research interest, which contains a self-inactivating (SIN) lentiviral LTR configuration where the homologous promoter/enhancer sequences in the U3 region of 3′ LTR are deleted. Thus, during reverse transcription, the deletion is reproduced in the 5′ LTR, reducing the risk of the activation of genes neighboring the LV insertion site and decreasing the risk of LV mobilization by HIV [7,22,28].

Recently, a fourth-generation LV candidate named LTR1 has been developed. This new LV has been designed to be safer than, and as productive as, the third generation. For this purpose, LTR1 reduces its portion of the wild-type HIV-1 genome to 441 bp. It also prevents the transfer of HIV-1 packaging sequences to patient cells by relocating them, which reduces the propensity to splice into target cell genes, enhancing safety. After transduction, cells start to express transgenes quicker than the third generation, with a similar titer yield [29]. LTR1 is a very promising successor to third-generation LVs, and we could consider its use in LV production.

As of now, more than 100 clinical trials are being carried out involving LVs, of which 25 started in September 2019 (Table 1) [30]. Two lentivirus-based ex vivo gene therapies have been approved in recent years. In 2017, the FDA-approved Kymriah (Novartis) [31], which is based on a SIN LV CD19-directed genetically modified autologous T cell immunotherapy, indicated for the treatment of patients up to 25 years of age with B-cell precursor acute lymphoblastic leukemia (ALL) that is refractory or in second or later relapse. Shortly after, in 2019, the EMA-approved Zynteglo (bluebird bio) [32], a SIN LV carrying a modified β-globin gene, which is added ex vivo to an autologous CD34^+^ cell population that contains hematopoietic stem cells (HSCs). After modified cell selection and amplification, they are reinfused into the patient for treating beta-thalassemia.

Large-scale LV production under current good manufacturing practice (cGMP) guidelines is necessary and challenging in gene therapy [33,34]. In general, the LV dose–response effect should be evaluated. The selection of the dose should be based on the findings obtained in the quality, nonclinical development of the product, and should be linked to its potency. An absolute number of functional lentiviral particles is not established; it will be determined by the gene defect/disease, the target tissue/organ, whether it is an ex vivo or in vivo approach, as well as the number of receptors on the target cells. When a classical dose finding is not possible, a minimal effective dose and maximum tolerable dose may provide useful information on the relationship between exposure and effect. Furthermore, it is known that for the phase I clinical trial, a large number of functional LV particles is needed.

There are currently many parameters being improved. Choosing the right packaging cell, vector transfection reagent, cell culture expansion process, or filtration and purification step directly affects the yield of the vector production. It is necessary for LV production to become more efficient and reach the required infectious unit number clinically needed more easily and quickly.

**Figure 2:**
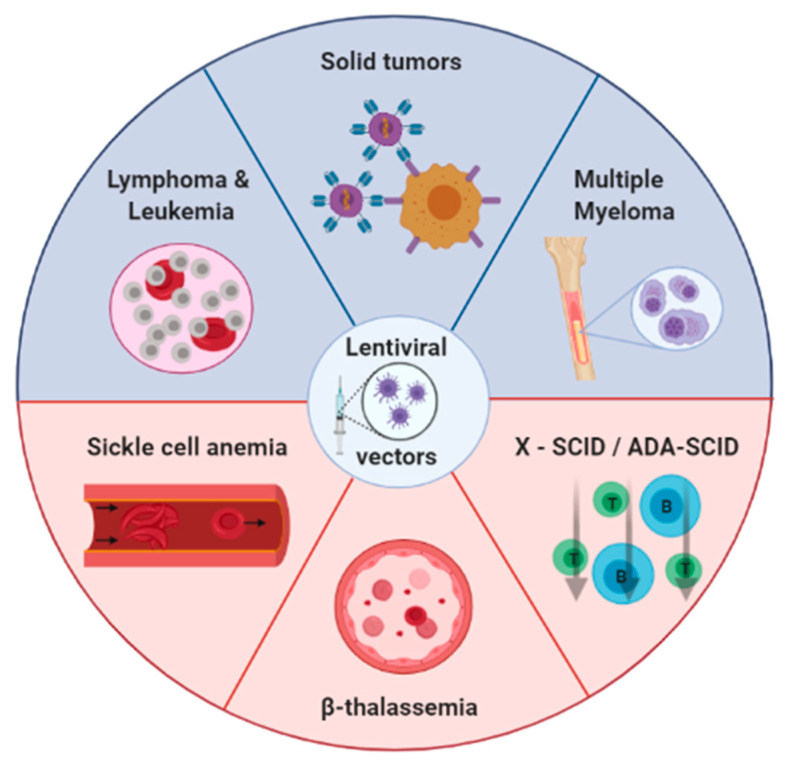
Main diseases with the highest number of active clinical trials using lentiviral vectors up to September 2020.

There are many devices and strategies to obtain the desired number of viral vectors for specific clinical or basic research. Depending on the researcher’s purpose, it is important to choose the appropriate method for LV production. Figure 2 shows the most studied diseases using LV-like β-thalassemia [35], sickle cell anemia [36], Wiskott–Aldrich syndrome [37], multiple myeloma [38], solid tumors [39], and COVID-19 [40], among others [41,42]. Here, we review the most effective ways to improve LV production.

## 3. Lentiviral Vectors Production

**Figure 3:**
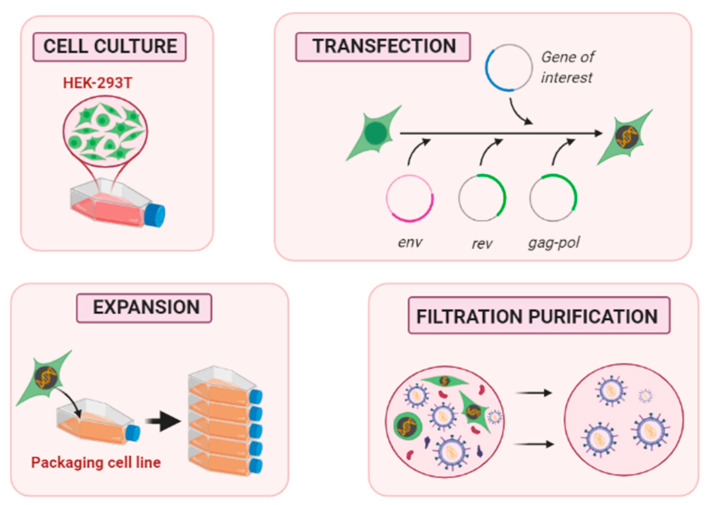
Proposed steps for lentiviral vector production in a packaging cell line.

Obtaining transducible viral vectors requires several steps (Figure 3). The first is to culture and expand a packaging cell line (PCL) to obtain a large enough cell population. The human embryonic kidney 293 cell line (HEK293) is traditionally used for efficient vector production. HEK293 is easily transfectable and adaptable to different culture strategies, which makes it ideal for LV production [43].

The next step is the transfection of plasmids into the packaging cells, which will produce vector particles. As previously mentioned, the strategy to prevent recombination is to divide plasmids into three or four separate transcription units, making it necessary to transfect different plasmids in the same packaging cell for expressing all required viral sequences at high levels [22]. Transient transfection is the easiest approach to obtain viral particles from a packaging cell, but it is possible to manufacture a stable PCL that generates viral vectors for larger periods of time. As we explain below, this is time-consuming, but it has some advantages from transient transfection [44]. After the transfected PCL is developed, it is necessary to expand the cell population to produce a sufficient number of viral particles. Titers of LV critically depend on the method of titration used. The titer can be estimated by measuring the number of viral particles capable of infecting cells efficiently, which we refer to as transducing units (TUs). Flow cytometry and quantitative polymerase chain reaction (qPCR) can be used for LV quantification of the TUs. In this case, the titer obtained only includes those particles that are able to transduce cells, and it is therefore important to include standard controls with the samples to standardize the titers from one experiment to another.

Finally, a purification and filtration step is crucial before collecting the product. The administration of a partially purified batch in animal studies, as well as human clinical trials, could generate toxicity or immunogenicity in patients [45].

### 3.1. Upstream Process

#### 3.1.1. Packaging Cell Line Culture

For products based on viral vectors, it is important to use packaging cell lines with no or minimal sequence homology with the vector and minimize the vector sequence homology with any human pathogens or endogenous viruses, thus reducing the risk of generating a novel infectious agent or replication-competent virus (RCV). Transient expression of packaging plasmids is a rapid and easy process for generating LVs. However, it is expensive, often inefficient, and prone to loss of gene expression [44]. Establishing stable, high-yielding recombinant cell lines remains the primary goal of protein manufacturers [46]. A stable PCL would ease large-scale LV production and its application in clinical trials [22]. However, these methods can be time-consuming (often taking several months), costly, and tedious. It is challenging to make a PCL that expresses all plasmids needed for LV production. In addition, the expression of some LV proteins, such as HIV-1 gag-pol, Rev, and the VSV-G attachment protein, are toxic for packaging cells, hindering the establishment of the PCL. Nonetheless, a stable PCL would enable harvesting several supernatants from a single-cell culture and the prospect of an inexhaustible LV producer.

There are two ways of establishing a stable PCL: through inducible or constitutive cell lines.

##### Constitutive PCL

Constitutive PCLs are based on replacing the VSV-G envelope to avoid cytotoxicity. Constitutive PCLs are more difficult and time-consuming to produce than inducible ones [47]. Only a few constitutive PCLs have been developed expressing all the LV plasmids. It has been challenging to find a delivery method for integrating the genes that encodes the LV plasmids while reducing cytotoxicity. Nevertheless, researchers are obtaining promising results in this field [48].

In 2003, the first stable PCL capable of expressing all the LV components was released. These PCLs were called STAR cells and were stable for several months with a titer of 10^7^ TU/mL. STAR cells were developed for expressing gammaretrovirus RD114 envelope proteins that are not cytotoxic. In addition, RV transduction was used for HIV *gag-pol* and *rev* integration and expression into the cell genome. It could generate replication-competent lentivirus, which affects safety [47].

The WinPac cell line was presented in 2015, avoiding the need for gammaretrovirus for plasmid integration [34]. During WinPac cell line production, an extra step removes gammaretroviral sequences through a cassette exchange. In spite of the more laborious method, after nine days of antibiotic selection, which ensures that most cells express all functions linked to the respective antibiotic resistance genes, WinPack cells can produce up to 5 × 10^6^ TU/mL and express LVs for four and five months in the presence or absence of antibiotics, respectively. It is adaptable to HYPERflask, enhancing production efficiency [34].

RD-MolPack cells were developed in 2013 [49] and enhanced in 2016 [50]. For HIV-1 *gag-pol*, *rev*, and hygromycin gene resistance transfection, it used a recombinant hybrid baculo-AAV vector on the HEK293T cell line, generating a “PK-7 clone”. The addition of the nontoxic SIN RD114-TR envelope and *tat* gene to the PK-7 clone generates RD2 or RD3-MolPack depending on the use of a second- or third-generation LV. Both present high stability and can get a production of 10^6^ TU/mL [49,50].

Tomás et al. recently released another constitutive PCL called LentiPro26 [51]. LentiPro26 has a SIN LV genome, and the additional plasmids have antibiotic resistance genes inserted, making this stable PCL very safe. LentiPro26 reduced the PCL generation time to 15 weeks by chemically transfecting HEK293T cells, using its antibiotic resistance for multiple selection steps. LentiPro26 is capable of producing up to 1.6 × 10^6^ TU/mL/d for more than 60 days and is scalable to HYPERflask, enabling harvesting 3.4 L of the supernatant [51]. This feature makes LentiPro a very promising and stable PCL for high-scale LV production.

##### Inducible PCL

These cell lines have VSV-G envelope proteins that are cytotoxic for cells, but its expression is induced only when LV production is required. It is usually made by a Tet system that consists of the repression of the transcription of HIV components. The tetracycline response element (TRE), present in the LV producer cell genome, reacts to the addition of a tetracycline/doxycycline antibiotic, inducing VSV-G expression and leading the LV production. These transcription control methods are made to avoid cytotoxicity, and many cell lines have been developed with this method [52,53], such as the stable PCL ProSavin, which is capable of producing high titers (up to 3 × 10^5^ TU/mL) for 83 days [54,55], or the one developed by Milani et al., which reached up to 4.4 × 10^6^ TU/mL [56].

One of the last inducible PCLs established is Clone 92. This cell line is stable, serum-free, and adapted to suspension. It was developed by Manceur et al. using the PCL described by Broussau et al. [54]. To avoid cytotoxicity, Clone 92 has a double switch system of tetracycline and cumate to induce transcription of *rev* and *VSV-G*, which is necessary to induce LV synthesis. *gag-pol* expression depends on the presence of *rev*. Clone 92 was optimized in shake flasks, obtaining 3 × 10^6^ TU/mL of LV titers, and was scalable to 3 L bioreactors with a perfusion mode, obtaining up to 8 × 10^10^ TU/L of bioreactor culture [57].

In summary, most current methods of LV production involve the cotransfection of preferably HEK293T cells because the presence of the SV40 T antigen in the producer cells makes them more efficient for vector production [57]. Furthermore, HEK293T cells show higher cell growth and transfection efficiency compared to others (e.g., HEK293) and can be adapted to suspension growth in serum-free medium, which is of particular interest for large-scale vector productions. Developing stable PCLs is potentially too time-consuming and costly for some researchers, who need high-scale LV production in a reduced lapse of time. However, in our opinion, the benefits of a stable versus transient PCL make the first more suitable for clinical use due to its reduced immunogenicity. The elimination of VSV-G protein in stable PCL reduces cytotoxicity, even if Gag, Pol and Rev are still toxic to cells. With inducible PCLs, it is possible to reduce the cytotoxicity of VSV-G, Gag, Pol, and Rev, obtaining a very safe cell line. Moreover, it is possible to design stable PCLs to grow in suspension or adapt them to bioreactors.

#### 3.1.2. Methods for Gene Transfection

There are many methods for virus plasmid transfer, generally divided into chemical and physical methods. High transfection efficiency is essential to generate high titer viral vectors.

For more than four decades, calcium phosphate (CaPO_4_) precipitation has been used in gene delivery and transient transfection for LV production [58,59], and is still currently being used. CaPO_4_ is an inexpensive method that can be used on several cell types, allowing the transfection of large amounts of cells at the same time. Despite CaPO_4_ not being the best option for large-scale LV production, since it is highly sensitive to pH variations and their cytotoxic effect on cells, it is suitable for producing LVs for animal studies. To avoid cytotoxicity, it is necessary to culture cells with serum or albumin and change culture medium after transfection [60].

On the other hand, polyethylenimine (PEI)-mediated transfection is another method to transfer plasmids. It is as effective as CaPO_4_. PEI presents toxic effects on cells, and it is important to choose an appropriate PEI/DNA ratio. Nonetheless, concern about media change after transfection or pH variation is not considered, unlike when using CaPO_4_. It is effective in adherent and suspension cultures, and serum is not indispensable [61]. All these features make PEI transfection more suitable when considering large-scale manufacturing of LV [62].

Lipofectamine (Invitrogen) is one of the most effective and easiest transfection reagents for generating LVs. Lipofectamine allows us to transfer many cell types and can transfer more than 70% of cells in a few days. Some protocols have reached almost 100% of transfected cells using lentivirus [63]. Although it is probably the best method for small-scale use, it is too expensive for large-scale production.

Physical methods, such as flow electroporation (EP), have also been used for LV production. Flow EP is as effective as CaPO_4_, requires one-third less DNA quantity, and there are no toxic effects on cells as it does not need chemical reagents. Flow EP can be used in suspension cell lines in bioreactors to produce large-scale lentivirus batches without increasing the contaminant level [64].

The addition of sodium butyrate to the culture medium after plasmid transfection has been reported to increase the production of LV titers in adherent and suspension cell cultures [65], but not in stable cell lines [51].

The transfer method will depend on the cell line chosen for LV production and the research state. PEI transfection seems to be more suitable for adherent cell cultures. However, the advantages of flow EP transfer made this option the best in suspension cell cultures. CaPO_4_ and polifectamine are very useful as well, but its use is more suitable on a small-scale as it occurs in the early stages of LV production research. Polifectamine is the one most appropriate to achieve an efficient transfer, while researchers can focus on other parameters.

#### 3.1.3. How to Improve the Lentiviral Vectors Production

Once the transfection method is chosen, the next step for LV production is packaging cell expansion. Any optimization method is useless if the number of cells is inadequate. There are different expansion methods depending on the culture type, and the easiest is to increase the total surface area of the tissue culture vessels or use suspension cultures that require the use of nonadherent or suspension-adapted cells. Both adherent and nonadherent cell types are useful for LV production, but each has different features to consider. Here, we present the most used cell lines of each type and the ways to expand them.

##### Adherent Cells Culture

The HEK293 variant, HEK293T, is an adherent cell line normally cultured in a T-flask in 10% fetal bovine serum (FBS)-supplemented medium. The HEK293T cell line contains the integrated SV40 T antigen, which provides a higher LV titer yield than the parental cell line HEK293 [66]. The objective is to optimize the total number of PCL cells capable of being transfected to maximize the total yield of the LV vector that will express the therapeutic gene of interest. Traditionally, T-Flasks have been used for cell culture, but they cannot produce a LV titer yield that is required for gene therapy. The most apparent step for enhancing the titer yield is increasing the cell culture surface.

One of the easiest methods to enhance LV production is using multilayer vessels. The HYPERFlask^TM^ (Corning) has a total growing area of 1720 cm^2^ of a gas permeable surface, leading to CO_2_ and O_2_ exchange. The production of a single HYPERFlask, compared to ten 150 cm^2^ culture flasks for LV production, is about 10-fold higher, obtaining 1.1 ± 0.16 × 10^11^ of total TU in a HYPERFlask and 1.2 × 10^10^ TU in a T-150 Flask. TU/mL was also higher, up to 2.3 × 10^8^, probably due to an improved gas exchange [67].

Multilayer cell factories (CFs) are also used for this purpose. The cell factory system (Nunc, EasyFill, ThermoScientific, Waltham, MA, USA) is a clever solution for increasing LV production. It concerns multiple interconnected layers of up to 40. In the larger version, culture surface area is equivalent in those 170 T-150 flasks. Ten-layer cell factories were scaled-up for LV production to harvest 50 L of viral stock per batch. Inside a CF, HEK293T cells are seeded and transfected by a calcium phosphate method. After purification, it obtains 2 × 10^9^ TU/mL with a total of 6 × 10^11^ TU [68]. Rout-Pitt and McCarron [69] recently described a production and purification method to produce a VSV-G pseudotyped LV in 10-layer CFs using calcium phosphate coprecipitation, also adaptable to PEI transfection. After ultracentrifugation, they achieved a LV titer yield of 10^8^–10^9^ TU/mL [69].

HYPERflasks, CFs, and T-Flasks are still great options to consider in clinical-scale LV production (Table 2). All these vessels are still used by cGMP facilities to produce clinical-grade vector preparations. In fact, even the old roller bottle system has been used by Amgen to produce Imlygic, the first FDA-approved gene therapy product. In addition, 42-stack HYPERstacks are routinely used in cGMP for adherent cells or systems, such as iCellis, for cells grown on flakes or beads. Moreover, these systems are useful for ex vivo therapies, though they are limited, difficult to scale up, and insufficient for in vivo applications. For large- or industrial-scale viral vector production, scalable devices are needed.

For increasing culture surface even more on these 2D devices, the only way is to multiply the number of devices used at the same time. However, it is time-consuming and increases the personnel need. This situation changed with the development of bioreactors, especially fixed-bed bioreactors (Table 3).

iCELLis bioreactor technology (Pall Life Sciences, Hoegaarden, Belgium) is based on the use of a compact fixed-bed filled with microfiber macrocarriers. The main feature of iCELLis is the large surface area provided by thousands of polyethylene terephthalate (PET) fibers. iCELLis generates large-scale, high-viral vector titers under cGMP while measuring and recording pH, CO_2_, and O_2_ levels. The iCELLis bioreactor also provides an integrated perfusion system that allows the culture medium to be replenished while metabolites are removed and viral vectors collected.

iCELLis “nano” and “500” models provide up to 4 and 500 m^2^ of cell culture area, respectively. The cell growth surface provided by the iCELLis “500” model is significantly high since it is equivalent to 3000 HYPERFlasks.

The iCELLis nano bioreactor has been used for scaling-up the production of particles of clinical interest, such as retroviral [70], adenoviral [71], and adeno-associated viral vectors [72]. In 2018, an iCELLis nano-optimization study for LV production was published using HEK293T adherent cells. In this study, CaPO_2_ and PEI transfection methods were compared. The best results were obtained with PEI, supporting this method for large-scale transfections. Using 2.67 m^2^ low-compaction bioreactor cells showed better distribution than the 4 m^2^ cells. iCELLis nano has a 700 mL media volume, and its perfusion system needs 5 to 7 L, obtaining a product volume of 3 to 5 L. In this way, it was possible to obtain 4.85 × 10^8^ viral particles (vp)/cm^2^ and 3.63 × 10^5^ TU/cm^2^. Although the control flask yield/cm^2^ was higher than the bioreactor, in this run, it obtained 9.70 × 10^9^ TU in total [62]. The comparison between the number of physical particles (or vp) and functional particles (or TUs) shows the yield loss at filtration and concentration steps.

Although iCELLis 500 has been adapted to provide large batches of adenoviral vector titers [71], adapting iCELLis nano LV production to iCELLis 500 has been challenging. After promising results from Valkama et al. [62] in iCELLis nano, Leinonen et al. tried to optimize iCELLis 500 to obtain the herpes simplex virus thymidine kinase (HSV-TK) with LV to treat glioblastoma. Ganciclovir (GCV) is used as a prodrug to obtain a suicide effect in cells transfected with the HSV-tk. This system targets dividing tumor cells without affecting the nondividing ones (including neural cells and some terminally differentiated nondividing glial cells, among others) so that TK’s activity acts on blocking cellular genome replication during division of the actively dividing tumor cells [73]. This gene therapy with LV was used previously to treat glioblastoma using animal models [74]. A low-compaction, 333 m^2^ bioreactor was used for this purpose and showed promising results. While a 100 m^2^ bioreactor harvests 165 L, the 333 m^2^ bioreactor harvests only 165 L, which eases the purification and filtration steps. Finally, 4.35 × 10^15^ vp were produced and 2.03 × 10^11^ TU could be obtained. Despite it being a high-scale TU production, there is a significant difference in the vp number, probably due to the difficulty of cooling large volumes, which damages LVs [75]. These differences in the number of vp and TUs showed the need to improve the concentration and purification processes.

Only one year later, the same group published their finding on improvements to the downstream (DS) process at iCELLis 500 [76]. There are several methods for the DS process but almost no publications on large-scale DS processing. This new process involves several steps, including clarification, concentration, buffer exchange with tangential flow filtration, and anion-exchange chromatography purification. Using C8166, a human cell line derived from adult T cell leukemia–lymphoma patient very susceptible to lentivirus infection, it is possible to get 1.97 × 10^9^ TU/mL and a total production of 1.12 × 10^12^ TUs [76].

iCELLis is not the only fixed-bed bioreactor. Univercells (Gosselies, Belgium) recently brought a new automated single-use fixed-bed bioreactor: the Scale-X^TM^ bioreactor system. The Scale-X^TM^ bioreactor provides a range of 2.4 to 600 m^2^ of tightly spiral-wound, nonwoven polyethylene terephthalate fabric layers. There is a medium-scale version of the bioreactor with a range of 10 to 30 m^2^. It has only one compaction level, and cGMP manufacturing is not available yet but will be for the next year [77].

In 2019, a Scale-X hydro 2.4 m^2^ bioreactor was tested to produce LVs. A study was developed by an expert group in viral vector production with some experience in fixed-bed bioreactors, and Scale-X hydro was compared with the most relevant factory of LV production on this scale: iCELLis nano. They applied similar iCELLis parameters in the Scale-X bioreactor since the material where cells adhere is the same in both bioreactors. The Scale-X hydro bioreactor reaches titer yields as high as those in iCELLis nano or even better. A total of 2.4 × 10^10^ TU and 9.8 × 10^5^ TU/cm^2^ was reached in Scale-X hydro facing 1.3 × 10^10^ TU and 4.7 × 10^5^ TU/cm^2^ in iCELLis nano as controls were obtained, while viral particles per milliliter were similar in both bioreactors (up to 1.11 × 10^11^ vp/mL). Scale-X hydro was proven to be at least as efficient as iCELLis nano in media and glucose consumption [77].

Until now, the 600 m^2^ Scale-X nitro version has not been optimized for LV production, and if it could be scalable from iCELLis 500, it would reach very promising yields as using AV.

##### Suspension Cells Culture

Using the serum-dependent adherent HEK293T cell line results in expensive and difficult large-scale LV production processes under cGMP due to the requirement of large amounts of expendable materials, such as T-Flasks, animal serum, laboratory work personal, and time consumption.

To overcome those drawbacks, researchers began to report several strategies to adapt HEK293 cell culture to suspension culture conditions. In 2006, Segura et al. used a HEK293 variant, the HEK293E cell line, which expresses an Epstein–Barr virus (EBV) nuclear antigen-1. It was the first cell line used for LV production in suspension culture. They obtained a good LV yield using serum-free media, and demonstrated the scalability of the cell culture using a 3 L bioreactor [78]. Since then, several HEK293 variants have been adapted to suspension growth for LV production [79,80].

Shortly after Segura’s results, a cell line originally developed for the production of adenoviral vectors (HEK293SF-3F6) was optimized for LV production and adapted to a bioreactor similar to the one Segura et al. used. They attained a production of approximately 3.5 × 10^11^ total TU and 8 × 10^7^ TU/mL [65].

Bauler et al. [81] recently presented a cGMP-LV production system using a modified HEK293T cell line. They converted the HEK293T adherent cells to grow in suspension without FBS and designed the cell line as SJ293TS. SJ293TS proved to be a very stable cell line capable of producing up to 2 ± 0.4 × 10^9^ TU/mL. The converted cell line consumes less plasmid DNA and media than the parental HEK293T cell line [81]. Despite the fact that adherent cultures have monopolized LV production research, suspension cell culture seems to be as promising as the first ones.

Suspension cell cultures show several advantages compared to adherent ones as (i) their growth is limited by the concentration of cells in the medium, which allows easy scale-up; (ii) they are easier to passage; (iii) they do not require enzymatic or mechanical dissociation; (iv) they do not require a tissue-culture-treated vessel to be cultured, only agitation for adequate gas exchange; (v) most suspension cell lines grow in serum-free media, which reduces costs, eases downstream processing, and reduces the risk of contamination. Regarding the bioreactor topic, we presented the optimization of iCELLis and Scale-X bioreactors for adherent cell cultures. The main obstacle of bioreactors is scalability, though, in suspension cell cultures, this is not such a big limitation. In our opinion, the future of the large-scale production of LV is the implementation of suspension cells as a stable LV producer.

### 3.2. Downstream Processing of LV

**Figure 4:**
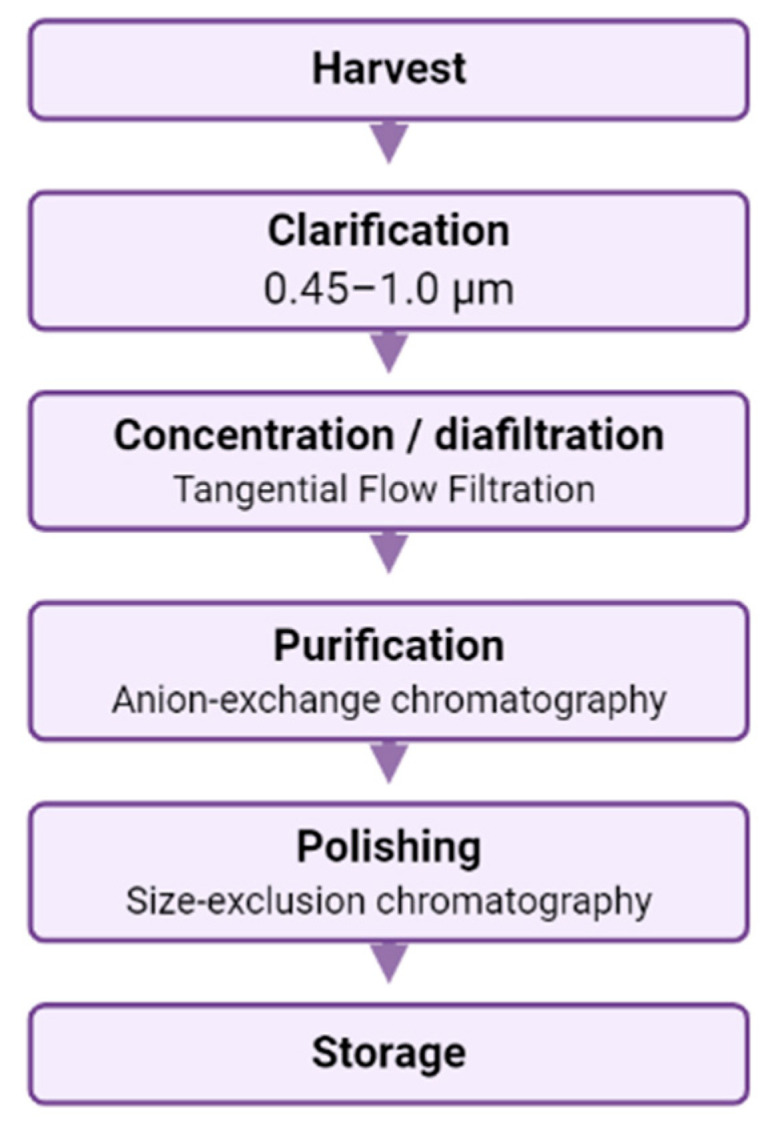
Flow chart of an example of downstream processing lentiviral vectors from harvested material.

After the production and harvesting of LV supernatants, the obtained product will be contaminated with some impurities, such as culture debris, plasmid DNA, cell-derived proteins, the PCLs themselves, product variants without enough efficacy or safety, and serum, the most contaminating product [82]. The DS process consists of a series of processing steps for removing all these impurities and concentrating harvest volume while LV activity is maintained (Figure 4). The major DS obstacles of LV have been handling large volumes and loss of functionality during processing. To maintain vector activity, short processing times and a reduced number of process steps are key. The methods and processing units used for small-scale concentration and purification are generally not applicable on a large scale.

The development of new large-scale LV production devices has increased cultures’ surface; however, the harvest volume has increased heavily by up to 165 L. For lentiviral vector-downstram processing, it is challenging to handle the concentration and purification of large volumes without losing functionality during the process. Furthermore, many of the purification–concentration methodologies, such as centrifugation, are not ideal for cGMP scale-up since, in cGMP, one produces very large-scale production runs and the total volume of media containing the LV product challenging under cGMP conditions. High-speed centrifugation tends to damage the virus envelope of LV or other enveloped vectors like HSV and measles. There are several reported methods adapted for large-scale DS processing, and we review the most used ones.

#### 3.2.1. Clarification

Immediately after vector harvest, the clarification process is carried out. Clarification consists of the removal of cell debris and packaging supernatant cells. Low-scale clarification was carried out by centrifugation and using microfiltration membranes with nanopores. For clarification of larger volumes, avoiding centrifugation and a single microfiltration step is preferred, and a 45 µm pore membrane was reported, though it seemed to produce pore obstruction with cells and debris, recovering up to 41% [83]. To avoid pore-clogging, the volume of supernatants passing per filter was reduced using a series of membranes with decreasing pore sizes, reaching more efficient filtration, reducing pore clogging, and obtaining better vector particle recovery of up to 66% [83]. Segura et al. used dual polysulfone membranes of 0.45–0.2 µm pores and a 500 cm^2^ filtration area to remove 2 L of cells and debris, reporting up to 93% infectious titer recovery [84]. Moreover, modern polyethersulfone membranes have proven to recover close to 100% of large-scale volume, clarifying 146 L/m^2^/h [76].

#### 3.2.2. Concentration and Purification

##### Tangential Flow Filtration

Tangential flow filtration (TFF) is an ultrafiltration method in which the previously clarified particles are concentrated and diafiltrated into a suitable buffer before chromatography. This process consists of the recirculation of supernatant volume through a membrane with small pore sizes between 1 and 100 nm [85].

TFF allows relevant volume reduction and viral particle concentrations in a short time and is easily adapted to cGMP manufacturing, making this method very suitable for LV concentration and ideal for large-scale supernatant processing. Unlike other ultrafiltration methods, TFF reduces membrane fouling, the main problem of ultrafiltration, which can reduce the flow rate [86].

TFF has proven to obtain recoveries between 90 and 100% of LV particles. This ultrafiltration method removes impurities, such as serum proteins, degraded DNA fragments, or transduction inhibitors, achieving, in some cases, vector recoveries of more than 100% of the product [86]. TFF has been adapted recently for filtration of volumes up to 100 L of LV product [76].

##### Chromatography

Chromatography is the major technique for the purification of biomolecules to be applied for human therapeutics. Chromatography is widely used for large-scale purification because it enables fast, efficient, and easily reproducible purifications. There are several chromatographic methods depending on the mechanism of separation.

Anion-exchange chromatography (AEX) is an efficient and cost-effective tool for LV purification. This technique is based on the positive charge of LV under neutral pH. Therefore, the viral vector supernatant passes through a column with a negatively charged matrix, and the positively charged particles bind to the matrix while all impurities pass through the column. AEX has been widely used for the purification of LVs [60,81,87,88], obtaining promising results with vector particle recoveries of up to 70%. Chromatography is easily scalable thanks to several column types and sizes and its reutilizable capacity, multiplying its binding surface. Recently, 11 kg of concentrated LV titers produced in iCELLis 500 was loaded into a 400 mL Sartobind Q (Sartorius) column for AEX, and despite the high loss due to the excess of product, 22.4% of the functional LV vector was recovered [76].

The viral vectors are exposed to high salt concentrations (0.5–1 M NaCl) to elute the bound particles from the anion-exchange columns. This is the main drawback of this technique, since high salt concentrations trigger the loss of viral activity. Just 1 h of exposure to 1 M NaCl at room temperature was enough to inactivate 50% of the virus [84].

Affinity chromatography is a purification technique based on the highly specific interaction between a target molecule and a ligand attached to a chromatography column for separating biomolecules. This technique leads to a reduction in the purification steps due to its high molecule selectivity [89].

There are several affinity chromatography techniques according to the interaction type: hydrogen bonds, electrostatic interactions, van der Waal forces, or antibody ligands, which are the most selective. However, using antibodies to interact with envelope proteins has not been described for retroviral or LVs. Heparin is an inexpensive affinity ligand that interacts with different virus types, including lentivirus. Heparin has been used for LV purification, recovering up to 53% of viral vectors and removing up to 94% of protein impurities and 56% of residual DNA [78,84]. NaCl is routinely used to elute vectors from heparin columns as the binding of vectors through their surface molecules is all based on charge–charge interactions of positively charged virus proteins to the negatively charged heparin column resin. Salt concentrations approaching 0.5 M are usually needed to eluate the virus from the column, and even this level of NaCl can be toxic to cells that would be infected with the purified vector. Thus, one needs to incorporate an additional polishing or purifying step to remove high salt concentration, such as using an additional TFF step that will enable buffer exchange [78,90]. It has been shown that heparin does not interact with the viral envelope proteins, leading to the interaction with different retroviruses types, without yield losses [91].

#### 3.2.3. Polishing and Storage

In large-scale purification protocols, size-exclusion chromatography (SEC) has been adopted as a polishing step, where this method is more suitable and efficiently removes remaining impurities. SEC, also known as gel filtration chromatography, is traditionally a purification method based on the larger size of viral particles compared to impurities. There is no molecule–ligand binding, so all the contaminants, which are smaller than the pore size, pass through the column, while larger viral particles are retained [86]. A 70% total of purified RV has been reported using SEC and more than 90% of purity. The main drawback of SEC is the difficulty to scale-up because of its low loading capacity (10% column volume). Furthermore, SEC purification requires low linear flow rates, which increase process time.

The short half-life of LVs makes its freezing after purification necessary. LV preparations are normally stored at −80 °C as there is no efficient formulation for storing them at 4 °C or room temperature. The half-lives of infectious particles between one and two days at room temperature and eight days at 4 °C have been reported [92]. However, storage at −80 °C leads to a number of functional units remaining after one and two years [76]. Before storage, it is necessary to change the buffer into a final cryoprotecting formulation. It has been reported that sucrose and magnesium chloride reduce the loss of infectious particles, increasing stability during storage [76,93,94]. In spite of this, some of the infectious particles are inactivated overnight when a large volume of media is frozen due to the long cooling time [94].

However, there is not only one correct strategy for downstream processing of LV preparations; different protocols can be made with the same techniques.

To ensure the quality of the final product and its safety for human use, it is necessary to obtain GMP certification. Several international agencies have released some guidelines for GMP in gene therapy, which consider every production step, used materials, personnel, equipment, etc. Focusing on product quality control, it is necessary to measure some specific parameters for checking vector purity, safety, or potency. ELISA assay is very useful to measure LV physical titers, residual benzonase, bovine serum, and residual host cell proteins. Quantitative PCR allows the detection of sequences of LV physical and functional titer, SV40, and plasmids and host cells, among others. There are many other methods to measure other parameters. These assays let us verify whether the obtained products are according to cGMP [95].

## 4. Conclusions

Since their development, LVs have become the most promising alternative to previously established viral vectors for gene therapy due to several advantages. The potential of LVs has been demonstrated with two gene therapies approved for clinical use since 2017. However, the large number of LV titers required for clinical trials and great difficulty of scaling-up these vectors remain a challenge.

It is becoming increasingly clear that upstream process optimization and scaling-up is required before downstream optimization. This will provide consistent feedstock availability for downstream development, ensuring repeatability.

Efforts are made in developing stable cell cultures. Despite the difficulty of developing these cell lines, most of the actual drawbacks in lentiviral production would be solved. A stable cell line would delete some of the steps that are needed in transient-transfection-based cell cultures, and these cells would produce vector particles constantly over a long time. Therefore, this cell culture would be a kind of lentiviral vector factory.

Two-dimensional (2D) devices for LV production have reached their production limit, and they can be difficult to optimize for a higher yield. Nonetheless, we are now at a large-scale production revolution. In only a few years, we have witnessed the enhancements of adherent packaging cell cultures, transient transfection methods, and new 3D culture devices as bioreactors. These new bioreactors have gained popularity, and researchers are optimizing them for large-scale lentivirus production. Adapting suspension cell lines to these bioreactors seems to be an attractive option for this purpose. These bioreactors generate larger supernatant volumes, which caused problems in downstream processing, but researchers are currently developing new strategies to overcome this drawback.

Thanks to progress in this field, each year, the production yield increases. We are convinced that in the coming years, large-scale lentiviral vector production will no longer be a challenging step and ease the applicability of lentivirus-based gene therapies.

## Figures and Tables

**Table 1:** Current LV-based clinical trials available from “clinicaltrials.gov” from September 2019 to September 2020 using the terms “Lentiviral vectors” as keywords.

Disease	Intervention	Reference	Phase
X-linked severe combined immunodeficiency (SCID-X1)	Transfer IL2RG complementary DNA (ex vivo)	NCT04286815	I
Mucopolysaccharidosis type IIIA	CD34^+^ cells expressing the human SGSH gene (ex vivo)	NCT04201405	I, II
Metastatic breast cancer	CAR T cells specific for a cleaved form of MUC1 (ex vivo)	NCT04020575	I
Fanconi anemia subtype A	CD34^+^ cells expressing the FANCA gene (ex vivo)	NCT04069533	II
Sickle cell disease/anemia	CD34^+^ cells expressing the βAS3 globin gene (ex vivo)	NCT03964792	I, II
	CD34^+^ cells expressing human γ-globinG16D and short-hairpin RNA734 (ex vivo)	NCT04091737	I
	CD34^+^ cells expressing LentiGlobin BB305 Drug Product (ex vivo)	NCT04293185	III
Non-Hodgkin’s lymphoma	Autologous CD19-directed chimeric antigen receptor (CAR) T cells (ex vivo)	NCT03938987	I, II
	Infusion of UCD19 CAR T cells (ex vivo)	NCT04240808	I
	CD22 CAR T cells (ex vivo)	NCT04088890	I
Hemophilia A	CD34^+^ cells expressing the B domain deleted from of human coagulation factor VIII (ex vivo)	NCT03818763	I
	CD34^+^ cells expressing human factor VIII gene (ex vivo)	NCT04418414	I
	CD34^+^ cells expressing a functional FVIII gene to overcome human clotting FVIII gene defect (ex vivo)	NCT03217032	I
COVID-19	Use LV to modify artificial antigen-presenting cells to express SARS-CoV-2 proteins and immunomodulatory genes and activate T cells (in vivo)	NCT04299724	I
	Use LV to express SARS-CoV-2 proteins and immunomodulatory genes to modify dendritic cells and to activate T cells (in vivo)	NCT04276896	I, II
Metachromatic leukodystrophy	CD34^+^ cells expressing the human arylsulfatase A gene (ex vivo)	NCT04283227	III
Pyruvate kinase deficiency	CD34^+^ cells expressing a correct copy of the deficient PKD gene (ex vivo)	NCT04105166	I
Hemophilia B	LV to deliver a functional FIX gene to overcome human clotting FIX gene defect (ex vivo)	NCT03961243	I
CD19 negative B-cell malignancies	Gene-modified T cells targeting CD19-negative B malignancies (ex vivo)	NCT04430530	I, II
Hematological malignancy	Genetically engineered NK cells (ex vivo)	NCT04093622	-
Hepatitis C	Immunotherapy (HCVax™) (in vivo)	NCT04318379	I
Multiple myeloma	C-CAR088 Drug (ex vivo)	NCT04295018	I
	CD4^+^ chimeric antigen receptor immunotherapy (ex vivo)	NCT04162340	II, III
	CD4^+^ chimeric antigen receptor immunotherapy (ex vivo)	NCT04162353	I
B-Cell lymphoma	CD22 CAR T cells (ex vivo)	NCT04088864	I

**Table 2:** Most-used 2D devices used for LV production.

Device		Surface	Yield	Total Production
T-150 Flask	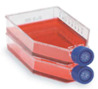	150 cm^2^	6.9 × 10^7^ TU/mL	1.2 × 10^10^ TU
HYPERFlask	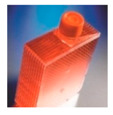	1720 cm^2^	2.3 × 10^8^ TU/mL	1.26 × 10^11^ TU
Multilayer Cell Factory	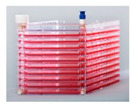	6320 cm^2^	2 × 10^9^ TU/mL	6 × 10^11^ TU

**Table 3:** Fixed-bed bioreactors optimized for high-scale LV production.

Device	iCELLis Nano	Scale-X Hydro	iCELLis 500+	Scale-X Nitro
	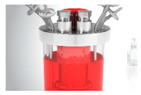	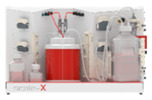	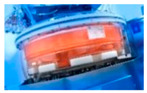	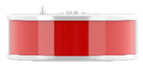
Surface	2.67 m^2^	2.4 m^2^	333 m^2^	200–600 m^2^
Total viral particles (vp)	>10^13^	>10^13^	9.27 × 10^14^	Not optimized yet
Vp/cm^2^	>10^9^	>10^9^	9.27 × 10^8^	Not optimized yet
Total transducing units (TU)	1.3 × 10^10^	2.4 × 10^10^	1.12 × 10^12^	Not optimized yet
TU/cm^2^	4.7 × 10^5^	9.8 × 10^5^	2.3 × 10^5^	Not optimized yet
Yield	Not concentrated	Not concentrated	1.97 × 10^9^ TU/mL	Not optimized yet

## Abbreviations

LV	Lentiviral vector
RV	Retroviral vector
FDA	Food and Drug Administration
EMA	European Medicine Agency
GMP	Good manufacturing practices
PCL	Packaging cell line
HIV	Human immunodeficiency virus
HEK	Human embryonic kidney
VSV-G	Vesicular stomatitis virus envelope glycoprotein
PEI	Polyethyleneimine
CF	Cell factory
